# Tissue Response to Subcutaneously Implanted Recombinant Spider Silk: An *in Vivo* Study

**DOI:** 10.3390/ma2041908

**Published:** 2009-11-20

**Authors:** Camilla Fredriksson, My Hedhammar, Ricardo Feinstein, Kerstin Nordling, Gunnar Kratz, Jan Johansson, Fredrik Huss, Anna Rising

**Affiliations:** 1 Laboratory for Experimental Plastic Surgery, Institution of Clinical and Experimental Medicine, Faculty of Health Science, Linköpings Universitet, 581 83 Linköping, Sweden; E-Mail: camilla.fredriksson@liu.se (C.F.); 2 Berzelius Clinical Research Center, Berzelius Science Park, 582 25 Linköping, Sweden; 3 Department of Anatomy, Physiology and Biochemistry, Swedish University of Agricultural Sciences, Box 575, the Biomedical Centre, 751 23 Uppsala, Sweden; E-Mail: my.hedhammar@afb.slu.se (M.H.); kerstin.nordling@afb.slu.se (K.N.); jan.johansson@afb.slu.se (J.J.); 4 National Veterinary Institute, 751 89 Uppsala, Sweden; E-Mail: ricardo.feinstein@sva.se (R.F.); 5 Department of Plastic-, Hand-, and Burn Surgery, University Hospital of Linköping, 581 85 Linköping, Sweden; E-Mail: gunnar.kratz@liu.se (G.K.); fredrik.huss@lio.se (F.H.)

**Keywords:** spider silk, recombinant, biocompatibility, *invivo*

## Abstract

Spider silk is an interesting biomaterial for medical applications. Recently, a method for production of recombinant spider silk protein (4RepCT) that forms macroscopic fibres in physiological solution was developed. Herein, 4RepCT and Mersilk^TM^ (control) fibres were implanted subcutaneously in rats for seven days, without any negative systemic or local reactions. The tissue response, characterised by infiltration of macrophages and multinucleated cells, was similar with both fibres, while only the 4RepCT-fibres supported ingrowth of fibroblasts and newly formed capillaries. This *in vivo* study indicates that 4RepCT-fibres are well tolerated and could be used for medical applications, e.g., tissue engineering.

## 1. Introduction

Tissue engineering is an interdisciplinary scientific field that applies the principles of engineering and life sciences to develop biological substitutes intended to maintain, restore, or improve tissue functions [1], and can arbitrarily be divided into two main principles:

- Autologous cells are cultured *in vitro* and transplanted back into the patient as a cell suspension, as a graft, or in a 3D-biodegradable carrier matrix.
- A tissue is stimulated and provided the right prerequisites to regenerate *in situ* by implantation of specially designed materials, or the application of substances that regulate cell functions. This method is often referred to as guided tissue regeneration.

In both these approaches biodegradable scaffolds are often used to facilitate the transplantation or ingrowth of cells and tissues [2,3,4,5]. As the scaffold temporarily replaces the tissue at target, the surrounding cells (or the cells transplanted with the matrix) migrate into (out of) the scaffold, proliferate, and subsequently form regenerated tissue. One crucial point is to use a scaffold with optimal qualities for e.g., cell adhesion, -migration, -proliferation, and -differentiation, as well as porosity, degradation, etc. Furthermore, a material intended for medical applications is obliged to be biocompatible, *i.e.,* elicit an appropriate host response reaction in a specific application without having toxic or injurious effects on the biological system. Also, the scaffold should allow and promote vascularisation. In recent decades, a number of materials have been approved for use as scaffolds, but still none can be considered ideal [3].

The bulk of scaffolds used today can be divided into synthetic polymers (e.g., polylactide/glycolic acids) and natural polymers (e.g., collagens, fibronectin, fibrin, silk). Of these, the natural polymers probably exhibit the best biomimetic properties [3,6]. However, collagen, fibronectin and fibrin are almost exclusively derived from natural sources, with the concomitant risk of residual antigens and/or infectious agents [3]. Also, the mechanical stability of these materials is not as good as synthetic polymers or silk [6]. Silk is a mechanically impressive proteinaceous fibre that is produced by e.g., silkworms and spiders [7,8]. Silkworm silk has been used as a suture material for centuries due to its mechanical properties and, more recently it has also been applied for tissue engineering purposes [9]. However, native silkworm silk contains a highly immunogenic protein coat (sericin) that causes hypersensitivity reactions [8]. Spider silk on the other hand, has no sericin coating and is known to be well tolerated when implanted [10,11], but has large scale production problems [12]. Spiders are difficult to farm and each individual produces small amounts of silk. Therefore, in order to gain large amounts, spider silk has to be produced recombinantly. Most heterologous expression systems available have been tested to produce spider silk. However, since the spider silk proteins are large, repetitive, and prone to aggregate [12,13,14,15,16,17,18,19,20,21,22,23,24,25,26], the success has been limited. Produced proteins have required the use of harsh solvents for solubilisation and subsequent spinning procedures to form fibres [14,16,21,22,23], or formed microscopic fibres intracellularly [20].

Recently, a successful strategy to produce recombinant spider silk protein, 4RepCT, in soluble form that self-assembles into macroscopic fibres was presented [27]. These fibres are as strong as mammalian tendons [27,28] and can be sterilised by autoclaving without change in properties (MH *et al*, to be published). Unlike many other man-made high performance materials, 4RepCT is produced at ambient temperature and pressure using only water as a solvent. In conclusion, 4RepCT is a novel protein-based material that has the potential to meet many of the desired features of implantable biomaterials, particularly for tissue engineering purposes. *In vivo* experimental analysis of tissue reactions to different substances is a valuable tool to investigate the safety and efficacy of a potential scaffold material. This study is the first to evaluate the tissue response to recombinant spider silk *in vivo*.

## 2. Results and Discussion

### 2.1. Materials

In this pilot study, the tissue response to recombinant spider silk (4RepCT) implanted subcutaneously in rats is evaluated. Each animal received six 4RepCT implants that had been subjected to different treatments (see experimental section and Table 1), one Mersilk^TM^ implant and one sham (*i.e.,* an incision is made without inserting an implant).

**Table 1:** Different treatments of 4RepCT before implantation. Abbreviations: Dulbecco’s Modified Eagle’s Medium (DMEM), and Fetal Bovine Serum (FBS).

Abbreviation	Treatment
C2	Two EndoTrap columns
C3	Three EndoTrap columns
C2M	Two EndoTrap columns, fibres soaked in DMEM
C3M	Three EndoTrap columns, fibres soaked in DMEM
C2MS	Two EndoTrap columns, fibres soaked in DMEM and FBS
C3MS	Three EndoTrap columns, fibres soaked in DMEM and FBS

Endotoxins are a natural part of Gram negative bacteria’s outer membrane, thus endotoxin contamination is hard to avoid in *Escherichia coli* production systems. These small and amphiphilic molecules may be hard to eliminate, and are known to be pyrogenic and potent activators of the innate immune system [29]. In this study, EndoTrap columns were used to remove endotoxins, and the fibres were subjected to an *in vitro* pyrogen test (IPT) [30,31]. The pyrogenicity of the C2 fibres (including a possible inherent pyrogenicity of the fibres themselves) was approximately 0.04–0.8 endotoxin units (EU)/mg (MH *et al*, to be published). Only C2-fibres were tested since these are expected to contain higher levels of endotoxin compared to the other 4RepCT-fibers. Since the 4RepCT fibres in this study weigh approximately 1.5 mg, the amount of endotoxins on the implanted fibres is well below the regulatory limit for medical devices (20 EU) [32]. However, since the IPT test only detects pyrogens exposed on the surface of the material, it is possible that endotoxins could be present in the fibre core. Such potential residual endotoxins could possibly be reduced by leaching (e.g., in DMEM), by removal before fibre formation by passage over an extra Endotrap column (C3), and/or shielded by deposition of plasma proteins on the surface of the fibre (by e.g., FBS). To investigate this, 4RepCT fibres were subjected to different combinations of these treatments (see experimental section and Table 1), and the results are discussed below.

**Figure 1:**
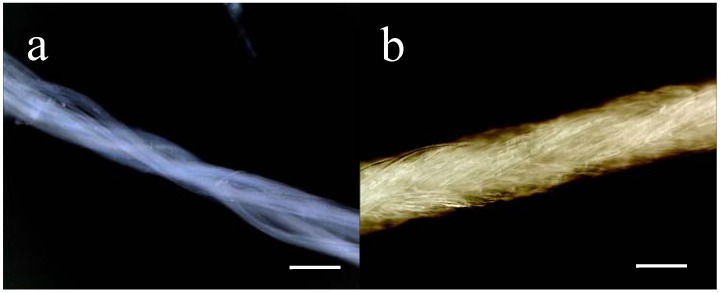
Macroscopic appearance 4RepCT fibres in (a) and of Mersilk^TM^ in (b). For illustration, an undyed Mersilk^TM^ suture (4-0, Ethicon) was used whereas dyed (black) sutures were used for implantation. Scale bars correspond to 0.1 mm.

As a control, Mersilk^TM^, made from silkworm silk and clinically used as suture material, was used. A comprehensive blinded study performed by Setzen and Williams comparing the tissue response to different suture-materials, among these silkworm silk, Vicryl^TM^ (polyglycolic acid) and PDS^TM^ (polydioxanone), showed that silk elicited an equivalent response to absorbable and non-absorbable materials, when counting the number of foreign body cells surrounding each suture-material [33]. Furthermore, they found a significantly higher response to multifilament sutures than monofilaments, confirming the influence of surface area-to-volume ratio in induction of an inflammatory response. In the present study, care was taken to compare two proteinaceous fibres of similar structure (Figure 1).

### 2.2. The normal tissue response to implanted materials

Host reactions following implantation of biomaterials include injury, blood-material interactions, and acute inflammation, followed by a foreign body reaction characterized by chronic inflammation, granulation tissue development, and fibrosis/fibrous capsule development [34]. The acute reaction is characterized at the histological level by the presence of polymorphonuclear leukocytes and macrophages, and usually resolves within one week. However, if the material persists in the tissue, the inflammation becomes chronic. The number of polymorphs may decrease and the presence of mononuclear cells (most commonly monocytes, lymphocytes and plasma cells) becomes more prominent, together with macrophages and multinucleated giant cells present at the implant surface. With biocompatible materials the chronic inflammatory phase lasts no longer than two weeks, even though the foreign body reaction at the tissue/material interface is present for the lifetime of the implant [34]. In addition to the acute and chronic inflammatory reactions, granulation tissue forms and is identified by the presence of macrophages, infiltration of fibroblasts and neovascularization of the healing tissue. In the present study, the animals carried the implants for seven days, which allowed for macroscopic evaluation of the acute inflammatory response and also histological evaluation of the tissue at the transition from acute to chronic inflammation.

### 2.3. Macroscopic evaluation

During the course of the study, all rats remained in good health and gained weight as expected (Supplementary Table 1). The recovery from the anaesthesia was rapid and the dermal incisions healed normally. The sites of implantation were inspected daily, and no inflammatory signs were noted.

**Figure 2:**
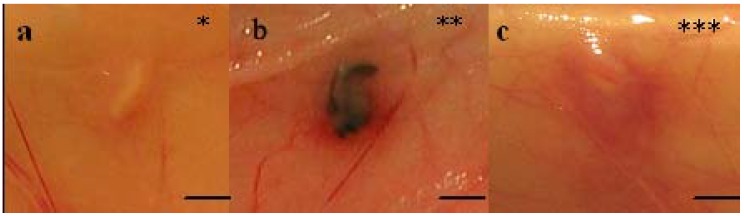
*In vivo* macroscopic appearances (graded * to ***) of implants at explantation. The rating in Table 2 is exemplified by (A) 4RepCT, degree* (C3 from animal no 1), (B) Mersilk^TM^, degree ** (from animal no 3), and (C) 4RepCT, degree*** (C3M from animal no 1). Scale bars are 1.0 cm.

At explantation, after seven days, the implants were inspected *in situ* before being removed (Figure 2a-c, Table 2). Five of the eighteen 4RepCT implants showed a local erythematous reaction surrounding the implants (Figure 2c), while the tissue surrounding five other 4RepCT implants and two Mersilk^TM^ implants showed no redness (Figure 2a, Table 2). The remaining eight 4RepCT implants and one Mersilk^TM^ implant showed an erythematous reaction at one pole of the implant (Figure 2b, Table 2). This reaction might be a part of the natural acute phase of the inflammatory reaction toward implanted biomaterials [34]. Two of the five implants that received the highest score for erythematous reaction (C3MS animal no. 1 and C2MS animal no. 3), later displayed local hemorrhage from an injured artery when examined histologically, which may explain the reddening. Apart from this, the reddening observed macroscopically could not be explained by inflammatory changes, as observed by the histopathological assessment (Table 2, Table 3).

### 2.4. Histological evaluation

**Figure 3:**
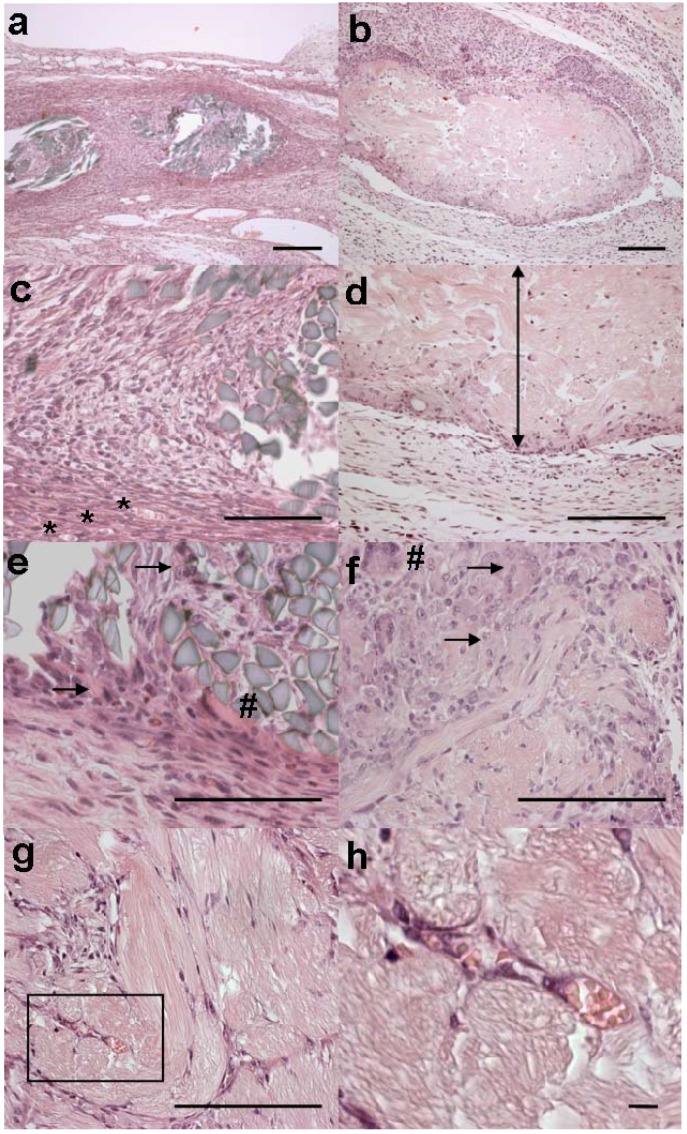
Microscopic appearances of implants. The Mersilk^TM^ implant from rat no. 3 is shown in (a), (c) and (e). 4RepCT fibre implants are shown in (b), (d) and (f) (C3 from rat no. 2), and in (g) and (h) (C2 from rat no. 2). Scale bars are 100 μm in a-g, and 10 μm in h.

Sections of the 4RepCT implants displayed subcutaneously located bundles of acidophilic fibres. The surrounding tissues were infiltrated by macrophages and multinucleated giant cells, the latter consistent with a foreign-body type reaction. Some implants showed a polar infiltration of cells, as exemplified in Figure 3b. In general, in the periphery of the 4RepCT implants, phagocytic cells, mainly macrophages but to some extent also multinucleated giant cells, displayed fibre-remnants intracellularly and increased cytoplasmic acidophilia. This suggests an ongoing degradation process, which was further corroborated by the presence of partially degraded fibres in the periphery of the implants, whereas the center of the fibre bundles seemed more intact. From the surrounding tissues, thin septae with delicate tubular structures of angioblast-like cells could be seen extending into some of the 4RepCT fibre bundles, consistent with formation of new capillaries. In line with this observation, erythrocytes were found in the lumen of these tubular structures (Figure 3h). Throughout the fibre-bundles, fibroblast-like elongated cells were found in close contact with individual 4RepCT fibres (Figure 3g). In most cases, a capsule of loose fibrous tissue, consisting of fibroblasts, fibrocytes, collagen fibres, and newly formed blood vessels, surrounded the 4RepCT fibre-bundle. In this capsule, some polymorphs and lymphocytes were occasionally observed (Table 3). These cells appeared to remain in the capsule or in the surrounding tissue rather than occupying the fibre-bundle.

The different preparations (double or triple EndoTrap purification, soaking in DMEM or in DMEM and FBS) of the 4RepCT fibres did not result in any obvious difference in host reaction or cellular response to the implants (Table 2, Table 3). However, additional and larger studies must be performed to draw any further conclusions. Two 4RepCT implants of eighteen could not be found at sectioning (Table 3).

**Table 2:** Macroscopic appearances (graded * to ***) of all implants (see also Figure 2). Implants where the surrounding tissue shows no rubor is indicated by *, slight rubor at one pole of the implant is indicated by **, and rubor surrounding the whole implant is indicated by ***, see Figure 2 for examples. The implants are listed in the left column and the numbers (1-3) in the top row indicate the different rats. Abbreviations; C2, C3, C2M, C3M, C2MS, C3MS corresponds to different 4RepCT fibre treatments (see experimental section and Table 1).

Implant	1	2	3
C2	*	**	**
C3	*	**	**
C2M	***	**	*
C3M	***	*	*
C2MS	***	**	***
C3MS	***	**	**
Mersilk^TM^	*	*	**
Sham	*	*	*

**Table 3:** Histopathologic assessment. The implants are listed to the left, for each individual. Abbreviations; C2, C3, C2M, C3M, C2MS, C3MS corresponds to the different 4RepCT fibre treatments (see experimental section and Table 1). Each implant has been rated according to the cell numbers/intensity of the lesions; +: low numbers of infiltrated leukocytes/mild changes. ++: intermediate numbers of infiltrated leukocytes/moderate changes. +++: numerous infiltrated leukocytes/severe changes. 0: not observed/ very low numbers of infiltrated leukocytes. nf: implant was not found at histological evaluation. -: Absence of implant (sham treated). * Phagocytic cells: macrophages and multinucleate giant-cells. ** Capsule: organized granulation tissues around fibres, including fibroblasts, fibrocytes, collagen fibres, angioblasts and newly formed capillaries. ^1^ Local haemorrhage.

	Polymorpho-nuclear cells	Phagocytic cells *	Mono-nuclear leukocytes	Capsule **	Granulation tissue between fibres
**Animal 1, implant**					
C2	+	+++	++	+++	+++
C3	+	++	+	++	++
C2M	+	++	+	+++	+
C3M	+	+++	++	+++	++
C2MS	+++	++	++	+++	+
C3MS	0	+++	++	+++^1^	+
Sham	-	-	-	-	-
Mersilk^TM^	+	+++	+	++	++
**Animal 2, implant**					
C2	0	+++	0	+++	+
C3	+	+++	+	+++	+
C2M	nf	nf	nf	nf	nf
C3M	0	++	0	++	++
C2MS	0	+++	+	+++	+
C3MS	0	+++	+	+++	++
Sham	-	-	-	-	-
Mersilk^TM^	+	++	0	+	+
**Animal 3, implant**					
C2	0	+++	++	+++	+++
C3	0	+++	+	+++	+++
C2M	0	+++	+	+++	+++
C3M	0	+	++	+++	+
C2MS	+	++	+++	+++^1^	+
C3MS	nf	nf	nf	nf	nf
Sham	-	-	-	-	-
Mersilk^TM^	0	+	+	+++	+++

**(a)** A fibrous capsule surrounds bundles of regular refringent fibres of the Mersilk^TM^ implant. **(b)** A polar infiltrate surrounds the 4RepCT implant, where one side evokes practically no response, at the bottom of the figure, while the opposite side, in the upper part of the figure, shows a moderate foreign body reaction with macrophages and multinucleated giant cells. **(c)** Capillaries are present in the fibrous capsule surrounding Mersilk^TM^ (*). Inside of the capsule, cells have populated the implant. **(d)** The side of the 4RepCT that evokes almost no inflammatory response is shown. The location of the 4RepCT implant is indicated by a double edged arrow. **(e)** The reaction in close contact with the Mersilk^TM^ implant displays macrophages (arrows) and multinucleated giant cells (#). **(f)** The side of the 4RepCT implant that evokes a moderate foreign body response is shown. The cell reaction adjacent the 4RepCT implant exhibit macrophages (arrows) and multinucleated giant cells (#). **(g)** The centre of a 4RepCT fibre bundle displays delicate septae with elongated cells, resembling fibroblasts, angioblasts and newly formed capillaries. The square box (magnified in h) encloses capillaries in the 4RepCT fibre bundle. **(h)** Magnification of square box in (g) shows erythrocytes in the lumen of tubular structures indicating capillaries present (and newly developed) in the centre of a 4RepCT fibre bundle.

The silkworm silk (Mersilk^TM^, dyed black by the manufacturer) fibres were refringent instead of acidophilic (Figure 3a). These fibres occupied less space in the tissue and appeared to be more intact than the 4RepCT implants. Essentially the same kinds and numbers of cells were found in the tissue surrounding the Mersilk^TM^ and 4RepCT (Table 3), but the phagocytes and macrophages adjacent to the fibres were smaller in size and the fibrotic capsule surrounding Mersilk^TM^ was more dense (Figure 3). In the Mersilk^TM^ fibre bundle, macrophages, multinucleated giant cells, and a few fibroblasts could be observed. However, in contrast to the 4RepCT implants, no newly formed capillaries could be identified and the infiltration of fibroblasts was much less pronounced in the center of the Mersilk^TM^ implants.

The sections that contained shams (*i.e.,* no implants) displayed changes in the subcutaneous tissue consistent with a normal wound healing reaction, *i.e.,* granulation tissue. Also, some focal areas of increased cell density (macrophages, multinucleated giant cells and polymorphonuclear cells) surrounding fragments of hair shafts were observed, consistent with foreign body type reaction.

The tissue surrounding both the 4RepCT and the Mersilk^TM^ implants were infiltrated by leukocytes, rich in macrophages and multinucleated giant cells (Figure 3 a-f). A fibrous capsule surrounded the implants, but appeared denser in the case of Mersilk^TM^ implants. Also, the tissue surrounding some implants contained polymorphonuclear cells (9 of 19 implants) and mononuclear leukocytes (16 of 19 implants), which is indicative of acute and chronic inflammation, respectively (Table 3). The histological analysis after one week implantation revealed numerous phagocytic cells, consistent with a foreign body reaction with features of both acute and chronic inflammation, which is the natural response to any implanted foreign object after one week [34]. Polyacrylamide gel (commonly used dermal filler) subcutaneously injected in rats, show a similar histological appearance with macrophages, polymorphonuclear leukocytes and a thin fibrous capsule after one week of implantation [35].

Angiogenesis may be an inherent effect of silk-based biomaterials [36,37], and in accordance, newly formed capillaries were observed in the tissue surrounding the 4RepCT and the Mersilk^TM^ implants. Neovascularization is a critical factor for successful tissue engineering since it is a prerequisite for a continuous support of oxygen, nutrients and biochemical cues to the cells in the three dimensional matrix [37]. Thus, the ability to support the formation of vascularised tissues is a desired feature for any material used for tissue engineering purposes. It has also been hypothesized that, like growth factors, any biomaterial able to stimulate angiogenesis without amplifying inflammation could be effective in stimulating wound healing [36]. The most pronounced histological difference between the 4RepCT and the Mersilk^TM^ implants was the presence of fibroblast-like cells and newly formed capillaries in the center of 4RepCT fibre bundles (Figure 3 g, h), indicating that the 4RepCT implants are superior in supporting (and perhaps even at promoting) the physiological migration of fibroblasts and angioblasts.

The 4RepCT fibres seemed to be degraded by macrophages, possibly by endocytosis and subsequent intracellular proteolysis. Degradation of Mersilk^TM^ fibres appeared to be more slow, but the presence of macrophages engulfing the fibres suggests a similar mechanism of degradation as for 4RepCT fibres. Mersilk^TM^ fibres are considered non-degradable since they retain at least 50% of their tensile strength after 60 days of implantation [8]. However, the silk fibres will eventually be degraded, probably by proteolytic degradation mediated by a foreign body response [8]. In general, implants that are degradable show a more intense immunological response than permanent materials [33].

### 2.5. Features of 4RepCT for tissue engineering

Successful tissue engineering requires a suitable material to use as scaffold. Many different materials (natural and synthetic, biodegradable and (semi)-permanent) have been employed, but most have proven suboptimal since they are unable to support the regenerating tissue in all aspects needed [3]. There are several potential advantages of using recombinant spider silk (4RepCT) as a biomaterial for tissue regeneration. First, 4RepCT fibres are mechanically robust and can support the formation of new tissue. Second as shown here, the material is biodegradable and will eventually be replaced by the host’s own tissue. Third, the three-dimensional structure and morphology of 4RepCT assemblies can be tailored to resemble the structure and morphology of the tissue to be replaced. Fourth, 4RepCT is recombinantly produced, reducing the risk of contaminating biohazardous components and allowing large scale production. Also, the results from this pilot study show that the 4RepCT is biocompatible when implanted subcutaneously *in vivo*.

## 3. Experimental Section

### 3.1. Production of 4RepCT 

The miniature spider silk protein (4RepCT) was produced in *E. coli* BL21(DE3) cells (Merck Biosciences) using a modified pET vector encoding the fusion protein His_6_/thioredoxin/His_6_/thrombin cleavage site/4RepCT, as previously described [38]. The cells were grown at +30 °C in kanamycin supplemented Luria-Bertani medium to an OD_600_ of ~1, induced with 0.5 mM isopropyl-β-d-thio-galactopyranoside, and further incubated for 2 hours at room temperature. Cells were harvested by centrifugation at 4,000 g and gently resuspended in 20 mM Tris, pH 8, and frozen at -20 °C.

### 3.2. Protein purification and fibre formation

After complete cell lysis with lysozyme and DNAseI, the 15,000 g supernatants were loaded on columns packed with Ni-sepharose (GE Healthcare, Uppsala, Sweden) and equilibrated with 20 mM Tris-HCl, pH 8.0. The columns were washed extensively (20 column volumes (CV)), first with 20 mM Tris-HCl, pH 8.0 and subsequently with 20 mM Tris-HCl, 30 mM imidazole, pH 8.0, before bound proteins were eluted with 300 mM imidazole. Pooled fractions were dialyzed against 20 mM Tris-HCl, pH 8.0 over night and 100 µM CaCl_2_ was added to the protein samples before being loading onto EndoTrap Blue columns (Profos AG, Regensburg, Germany). After the void volume was discarded, the flow-through was collected and diluted to a concentration of 1 mg/ml. 4RepCT was released from the tags by proteolytic cleavage using a thrombin:fusion protein ratio of 1:1,000 (w/w), and allowed to pass over an other Ni-sepharose column (GE Healthcare, Uppsala, Sweden) to remove the tags. The solution containing 4RepCT was subsequently allowed to pass one or two additional EndoTrap Blue columns before being concentrated in Amicon concentrators and was then allowed to self-assemble into fibres as previously described [27]. Each fibre was formed from 1.5 mg protein. Fibres were removed from the tubes and washed twice in 20 mM Tris-buffer and twice in sterile water in order to remove any traces of the soluble released tags. The fibres were autoclaved for 2 × 15 minutes at +121 °C and 2.8 bar in tubes filled with sterile water. The fibres were stored at +4 °C until used. 

### 3.3. Treatments of protein and fibres

Fibres were made from protein solutions that in total were allowed to pass over two (C2) or three (C3) Endotrap Blue columns. Subsequently the fibres were subjected to one of three different treatments, giving the 6 different fibres for implantation:

1. Two Endotrap columns, no additional treatment (C2)
2. Three Endotrap columns, no additional treatment (C3)
3. Two Endotrap columns, soaked in DMEM (Dulbecco’s Modified Eagle’s Medium, Invitrogen AB, Lidingö , Sweden) for 30 minutes prior to implantation (C2M)
4. Three Endotrap columns, soaked in DMEM for 30 minutes prior to implantation (C3M)
5. Two Endotrap columns, soaked in DMEM containing 10% fetal bovine serum (FBS, Invitrogen AB, Lidingö, Sweden) for 30 minutes prior to implantation (C2MS)
6. Three Endotrap columns, soaked in DMEM containing 10% FBS for 30 minutes prior to implantation (C3MS).

As control-implants, commercially available silkworm silk suture (Mersilk^TM^) was used (4-0, Perma-hand Seide, Ethicon, Somerville, New Jersey). The appearances of 4RepCT fibres and Mersilk^TM^ sutures are shown in Figure 1.

### 3.4. Pyrogen analysis 

The pyrogen content in protein solutions and fibres was measured using an in vitro pyrogen test (IPT) (MH, *et al.* to be published). Briefly, human whole blood was brought into direct contact with the 4RepCT fibres (C2) and the release of the pro-inflammatory cytokine IL-1β was measured [30,31]. Lipopolysaccharide (LPS) concentration-response curves were derived using LPS from *Salmonella abortus equi.*

### 3.5. Subcutaneous implantation in rats

The study was approved by the ethics committee of animal experiments (Linköpings Djurförsöksetiska Nämnd, Linköping, Sweden, Dnr 96-08). Three 8-week old female Wistar rats (*Rattus norwegicus*, Scanbur, Sollentuna Sweden), weighing 159-184 g were included in the study. The rats were housed at the animal unit at the University of Linköping, at 25 °C and on a 12-hour-light-dark cycle. The rats had free access to water and low-fat rodent pelleted diet and were allowed to acclimatize for one week prior to the experiment. The rats were weighed on a regular basis (see supplementary Table 1). During surgery (implantation of the study materials) the rats were anaesthetised by inhalation of isoflurane (Baxter Medical AB, Kista, Sweden) and given Temgesic^®^ 0.3 mg/mL (buprenofin 35 μg/kg, Schering-Plough AB, Stockholm, Sweden) by subcutaneous injection as analgesia. A subcutaneous injection of 2.5 mL physiological saline (9 mg/mL, Baxter Medical AB, Kista Sweden) ensured maintained fluid balance. The back of each rat was shaved and swabbed with iodine (0.1%, Pharmaxin Sweden AB, Helsingborg, Sweden) and then four sagital incisions (approximately 1 cm long) parallel to the midline were made on each side with a scalpel. By blunt dissection with scissors, approximately 1 cm wide subcutaneous pockets were formed. Each animal received seven implants (C2, C3, C2M, C3M, C2MS, C3MS and Mersilk^TM^) and one sham incision, randomly distributed. The implants were 5 cm long and approximately 0.15 mm in diameter (Figure 1), rolled up in a bundle. The animals were monitored for local symptoms at the wound area on a daily basis. Seven days after implantation the animals were euthanized by an intraperitoneal injection of 5 mL sodium penthobarbital (Penthobarbital natrium vet., 60 mg/mL, Apoteket Laboratory Products, Sweden).

### 3.6. Histological observations

The dorsal cutis and subcutis was removed from the underlying tissues, and implants with surrounding tissues were inspected macroscopically and photographed before samples were excised. After excision the implants with surrounding tissue were fixed in 10% buffered neutral formalin for 2 days, processed for histology and embedded in paraffin. Sections, 4 μm thick were cut with a microtome (Historange Microtome, LKB, Bromma, Sweden) and stained with haematoxylin and eosin (H&E) or van Gieson (VG) and mounted in Pertex^®^ (Histolab, Göteborg, Sweden). Stained sections were evaluated using a Nikon Eclipse E600 microscope and images were photographed using a Nikon DXM1200 digital camera (Nikon, Tokyo, Japan). The tissue sections were examined and scored blindly by a pathologist.

## 4. Conclusions

4RepCT fibres are well accepted when implanted subcutaneously in rats. In particular, the presence of newly formed capillaries and fibroblast-like cells in the center of the 4RepCT fibre-bundles already after one week implantation indicates that the 4RepCT fibres support the formation of vascularised tissue. Further *in vivo* studies are warranted to fully evaluate the ability of the 4RepCT fibres as a scaffold-material for tissue engineering in humans.

## Supplementary Materials

Supplementary materials can be downloaded online at http://www.mdpi.com/1996-1944/2/4/1908/s1/.
