# Supplementary material for: Tissue Response to Subcutaneously Implanted Recombinant Spider Silk: An in Vivo Study
Source: Materials (Basel). 2009 Nov 20;2(4):1908–22. doi: 10.3390/ma2041908 (PMC5513568; doi:10.3390/ma2041908)
Supplement: Supplementary file 1 [file materials-02-01908-s001.pdf]

Supplementary Materials

Tissue Response to Subcutaneously Implanted Recombinant Spider Silk: An *in Vivo* Study

Camilla Fredriksson [1,2], My Hedhammar [3], Ricardo Feinstein [4], Kerstin Nordling [3], Gunnar Kratz [5], Jan Johansson [3], Fredrik Huss [5] and Anna Rising [3,*]

Table 1. Weights (in grams) of animals included in the study. The individual animals (1-3) are listed to the left. Day 1 corresponds to the time of arrival, day 7 to the time of implantation (start of study) and day 14 to the day of explantation (end of study).

Animal	Day 1 (g)	Day 3 (g)	Day 5 (g)	Day 7 (g)	Day 9 (g)	Day 11 (g)	Day 14 (g)
**1**	143	151	158	159	180	200	201
**2**	148	165	175	182	234	257	258
**3**	154	168	177	184	228	253	259
